# Impact of diet change on the gut microbiome of common marmosets (*Callithrix jacchus*)

**DOI:** 10.1128/msystems.00108-24

**Published:** 2024-07-08

**Authors:** Cassandra Tang-Wing, Ipsita Mohanty, MacKenzie Bryant, Katherine Makowski, Daira Melendez, Pieter C. Dorrestein, Rob Knight, Andrés Mauricio Caraballo-Rodríguez, Celeste Allaband, Keith Jenné

**Affiliations:** 1 Animal Care Program, University of California, San Diego, La Jolla, California, USA; 2 Skaggs School of Pharmacy, University of California, San Diego, La Jolla, California, USA; 3 Department of Pediatrics, University of California, San Diego, La Jolla, California, USA; 4 Bioinformatics Graduate Program, University of California, San Diego, La Jolla, California, USA; 5 Center for Microbiome Innovation, University of California, San Diego, La Jolla, California, USA; 6 Department of Computer Science and Engineering, University of California, San Diego, La Jolla, California, USA; 7 Shu Chien-Gene Lay Department of Bioengineering, University of California, San Diego, La Jolla, California, USA; 8 Halıcıoğlu Data Science Institute, University of California, San Diego, La Jolla, California, USA; Max Planck Institute for Marine Microbiology, Bremen, Germany

**Keywords:** microbiome, metabolome, nutrition, physiology, metagenomics, veterinary microbiology, nonhuman microbiome, nonhuman microbiota, marmoset, primate

## Abstract

Gastrointestinal diseases are the most frequently reported clinical problems in captive common marmosets (*Callithrix jacchus*), often affecting the health and welfare of the animal and ultimately their use as a research subject. The microbiome has been shown to be intimately connected to diet and gastrointestinal health. Here, we use shotgun metagenomics and untargeted metabolomics in fecal samples of common marmosets collected before, during, and after a dietary transition from a biscuit to a gel diet. The overall health of marmosets, measured as weight recovery and reproductive outcome, improved after the diet transition. Moreover, each marmoset pair had significant shifts in the microbiome and metabolome after the diet transition. In general, we saw a decrease in *Escherichia coli* and *Prevotella* species and an increase in *Bifidobacterium* species. Untargeted metabolic profiles indicated that polyamine levels, specifically cadaverine and putrescine, were high after diet transition, suggesting either an increase in excretion or a decrease in intestinal reabsorption at the intestinal level. In conclusion, our data suggest that *Bifidobacterium* species could potentially be useful as probiotic supplements to the laboratory marmoset diet. Future studies with a larger sample size will be beneficial to show that this is consistent with the diet change.

**IMPORTANCE:**

Appropriate diet and health of the common marmoset in captivity are essential both for the welfare of the animal and to improve experimental outcomes. Our study shows that a gel diet compared to a biscuit diet improves the health of a marmoset colony, is linked to increases in *Bifidobacterium* species, and increases the removal of molecules associated with disease. The diet transition had an influence on the molecular changes at both the pair and time point group levels, but only at the pair level for the microbial changes. It appears to be more important which genes and functions present changed rather than specific microbes. Further studies are needed to identify specific components that should be considered when choosing an appropriate diet and additional supplementary foods, as well as to validate the benefits of providing probiotics. Probiotics containing *Bifidobacterium* species appear to be useful as probiotic supplements to the laboratory marmoset diet, but additional work is needed to validate these findings.

## INTRODUCTION

The common marmoset (species: *Callithrix jacchus,* family: Callitrichidae) is an increasingly prevalent New World non-human primate used in biomedical research due to their small size (300–450 g), early maturation, relatively short lifespan (10–12 years), and high fecundity (2–3 offspring every 5–6 months) (1, 2). They are a well-established animal model used for behavioral testing, neuroscience, aging, toxicology, infectious disease, obesity, and reproductive biology (1–4). In the forests of Brazil, common marmosets are omnivores and exudivores, with a diet that is primarily composed of plant exudate, insects, fruit, seeds, sap, flowers, and even small animals (5, 6). In the wild, marmosets primarily eat 45% exudates, 16% fruits, and 39% insects (7). In the laboratory setting, commercial diets are formulated as biscuit or gel diets and are provided as the main meal, appropriately meeting the requirements for vitamins and minerals. Specifically, marmosets do not make vitamin D3 in their bodies, and, therefore, commercial diets sufficiently provide this need. Captive marmosets should also be supplemented with a variety of other foods to replicate their natural diet, such as fresh and dried fruit, vegetables, seeds, and nuts, as well as protein from mealworms and insects (4). In a captive setting, there is a lack of agreement regarding the appropriate diet since little is known about the optimal husbandry and nutrient requirements (4). Captive diets vary between institutions, ranging from cafeteria style to commercial purified and irradiated diets or biscuit and gel diets, typically influenced by anecdotal experiences and animal preferences (4, 8, 9). Nutritional requirements and diet are key husbandry factors that can directly influence weight and health. This is especially true for gastrointestinal diseases, such as inflammatory bowel disease (IBD), chronic enteritis, and colitis, which are all prevalent issues in captive common marmoset colonies (8, 10). Underlying health conditions, such as GI diseases (chronic diarrhea, inflammatory bowel disease, and chronic lymphocytic enteritis) or metabolic dysfunctions, obesity, and stress in captivity, can contribute to unexplained experimental variations and even potentially negatively impact research outcomes (11, 12). Therefore, there is a critical need to further understand gut and nutritional requirements and to standardize dietary husbandry.

Studies of the marmoset microbiome are still in the early stages, yet there is increasing interest in understanding the role of the gut microbiome in the health of marmosets. In particular, the gut microbiome has been shown to be intimately connected to diet and gastrointestinal health (11, 12). Several studies have used 16S amplicon sequencing and quantitative PCR to show that the captive marmoset microbiome is dominated by *Bacteroidetes, Bifidobacterium, Proteobacteria, Fusobacteria*, and *Actinobacteria* (11–17). Other studies have found that captive marmosets’ guts were enriched in *Enterobacteriaceae*, while wild marmoset guts were enriched with *Bifidobacterium* (5, 18). *Bifidobacterium,* thought to process host indigestible carbohydrates, has been shown to be an important component of the gut microbiome since it is vital to the metabolism of tree gums, which are consumed by wild callitrichids (5, 13). Previous research has also shown that common marmosets are colonized from infancy and throughout their lives by a community of *Bifidobacterium* species with species-specific genomic content (5, 19). Since 2012, at least five novel species of *Bifidobacterium* have been described from the common marmoset feces (20–23). Therefore, further characterization of *Bifidobacterium* is necessary to decipher its role in the microbiome of the common marmoset.

Sex and age also influence the gut microbiome in group-housed marmosets (15, 17). Geriatric marmosets were found to have significantly altered microbiome composition, with a higher abundance of *Proteobacteria* and *Succinivibrionaceae* and a lower abundance of *Firmicutes* and *Porphyromonadaceae*, compared to young adult marmosets (15). When males and females were pair-housed to assess social behavior, males had greater compositional variation than females, and female gut microbial communities stabilized more gradually (17). While 16S amplicon sequencing has been used in many studies to assess the microbiome, the metabolome of the common marmoset is also being explored to characterize the health of the animal in a variety of settings. A few studies have assessed blood plasma using high-resolution metabolomics to understand changes during aging (24–26). However, further characterization of the metabolome is required to determine the effects of diet on the gut microbiome. Taken together, many studies have evaluated the microbiome and metabolome to integrate nutrition and metabolism, which we further explore here by using high-resolution metagenomic and metabolomic analyses.

**Fig 1:**
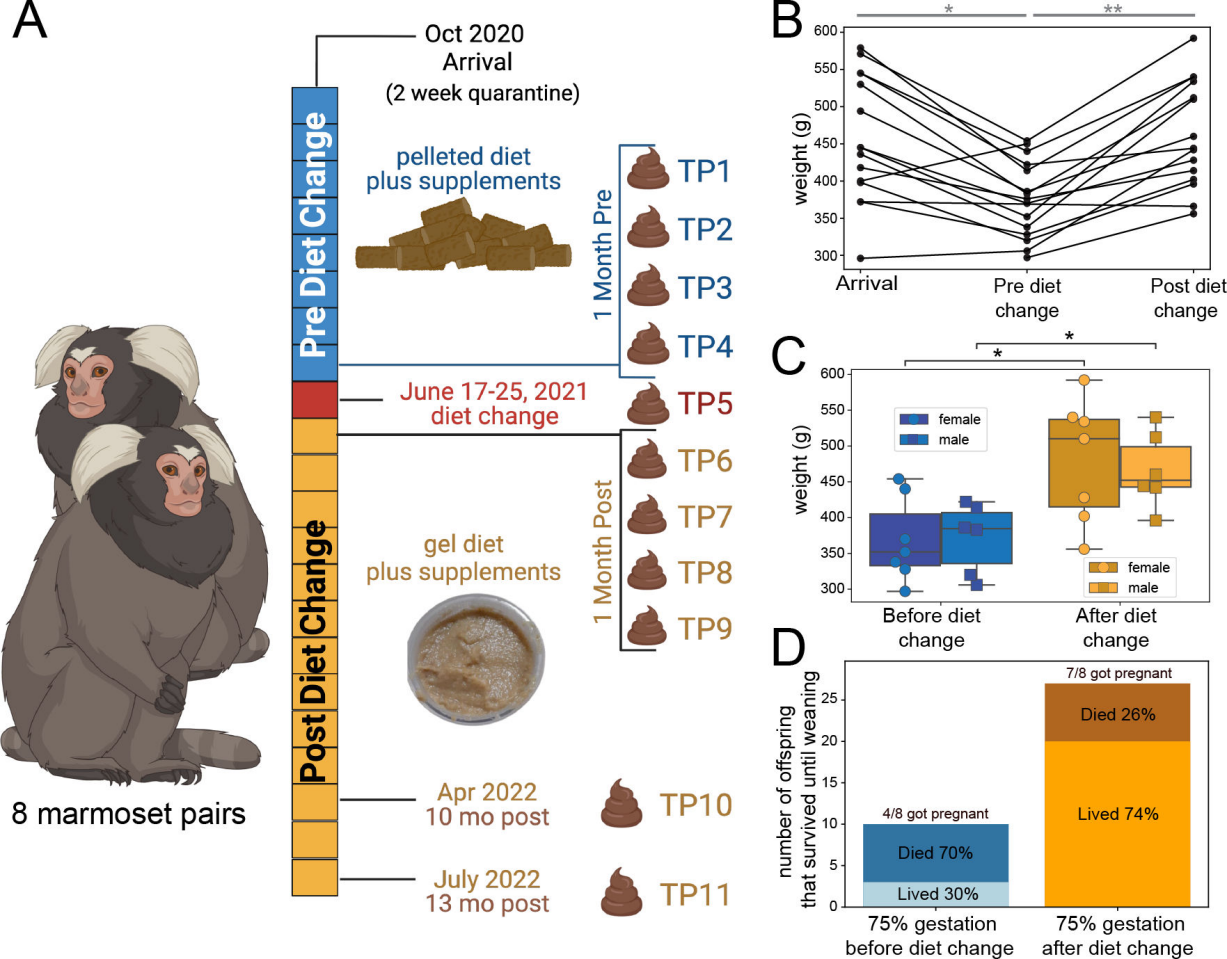
Diet change resulted in improved weights and reproduction. (**A**) Experimental overview: eight pairs of marmosets (1:1 males:females) pair housed (16 individuals) after quarantine period. They were fed a commercially processed Lab Diet 5040 (biscuit diet). Once weights and reproductive outcomes were noted to decline, a diet transition was made to an in-house prepared gel diet (Mazuri Callitrichid gel diet). Fecal samples were collected at four time points before, once during, and at four time points after the diet transition and then collected again at 10 months and 13 months post-diet change—a total of 11 time points. Created with BioRender.com. (**B**) Spaghetti plot of marmoset weights over time, from arrival (October 2020), 6 months after introduction and acclimation (April 2021; fed biscuit diet), and 1 year later (April 2022; fed gel diet). (**C**) Boxplot with swarmplot of marmoset weights, separated by sex, pre-diet change (April 2021; fed biscuit diet) compared to 1 year later, post-diet change (April 2022; fed gel diet). (**D**) Barplot of the number of babies born with at least 75% of gestation before (total of 7 months) and after (total of 13 months) the diet transition, sub-sectioned by how many of the offspring survived until weaning. Significance was determined by Mann-Whitney-Wilcoxon test two-sided with Bonferroni correction. ***P value < 0.001, **P value < 0.01, and *P value < 0.05.

Poor clinical health, including decreased weights and reproductive outcomes, was noted after a breeding colony was established at our facility. In particular, one adult female marmoset was observed to have significant weight loss, was markedly dehydrated, and did not become pregnant. Clinical blood chemistry values revealed mild anemia and moderate azotemia, suggestive of kidney issues possibly due to chronic dehydration. With concern for her health, she was transferred to another internal facility, where the marmosets were fed the gel diet. After the transfer, her weight, blood values, and overall health markedly improved. A few weeks later, an adult male marmoset from the same colony also had significant weight loss and marked dehydration. He was kept in the facility and switched to a gel diet based on the outcome of the previous case. He also showed marked clinical improvement after the diet switch. The remainder of the colony began to present with similar clinical symptoms, and the clinical decision was made to transition all individuals in the breeding colony from the biscuit to the gel diet for health. Both the biscuit diet, Lab Diet 5040 (Purina, Wayne County, IN, USA), and gel diet, Mazuri Callitrichid gel diet (Purina, Arden Hills, MN, USA), used at our facility are common commercial diets with comparable nutritional benefits, meeting the vitamin and mineral requirements for common marmosets. However, given that most research facilities either moisten their biscuit diets or combine them with other moist foods, like a gel or canned diet, and that our facility’s main colony of marmosets was already being fed the gel diet without these health concerns, it gave us further motivation to switch the colony to the gel diet. We hypothesized that the change in diet would result in a shift in the metabolites and microbes present in the gut, which would be beneficial to the health of the animals. We aimed to determine what makes the gel diet more beneficial than the biscuit diet and to use those findings to suggest supplements that can be ideal for captive common marmoset housed in a laboratory setting. Fecal samples were collected before, during, and after the diet transition. Fecal samples were collected from cage liners of paired marmosets at four time points in the month prior to the diet change, once during the transition, four time points in the month after the diet change, once 10 months post, and once 13 months after the diet change (Fig. 1A). Primary diet samples were also collected for metabolomics. Dietary supplements and treats (such as crickets, arabic gum, mealworms, yogurt, and marshmallows) were held constant pre- and post-diet change. Microbiome (shotgun metagenomics) and metabolome [untargeted liquid chromatography-tandem mass spectrometry (LC-MS/MS)] were used to evaluate the microbial and metabolic profiles of fecal samples over time. The overall analysis aimed to characterize the gut microbiome and metabolome of the colony of common marmosets to correlate the clinical improvements observed with the diet change.

## RESULTS

### Dietary switch from biscuit to a gel diet improved overall health and reproduction of common marmosets

Marmosets fed the biscuit diet displayed a significant decline in clinically observed health parameters including weight (Fig. 1B). After the transition to the gel diet, there was a significant improvement in observed clinical presentation and weight for both males and females (Fig. 1C; Fig. S1A). Additionally, there was a significant improvement in reproductive outcomes post-diet change (Fig. 1D). In the 8 months before the diet change, four of the eight adult female marmosets in the colony became pregnant and only 30% of their offspring survived. In contrast, after the diet change, seven of the eight adult female marmosets became pregnant and 74% of the offspring survived in the 13 months after the diet change. Therefore, transitioning from the biscuit to gel diet improved the overall health, weight, and reproductive outcomes in the common marmosets at UC San Diego’s breeding colony.

Facility of origin and increased weights during pregnancy may be confounding variables to these conclusions. Although there is not a significant sample size, there appears to be a better response in weight gain in marmosets that were originally transferred from an internal facility (pre- vs post-weights, *P* = 0.007, Mann-Whitney-Wilcoxon test two-sided) (Fig. S1B) compared to those transferred from an external facility (pre vs post, *P* = 0.1775, Mann-Whitney-Wilcoxon test two-sided) (Fig. S1C and D). However, less than half of the marmosets (6 [4 female, 2 male] out of 16) were transferred from multiple external facilities, so the sample size from each unique facility is small. Multiple external facilities were chosen in order to increase the genetic diversity of the breeding group. The diet provided by the separate external facilities also reportedly varied. Together, these variables make conclusions based on the facility of origin unclear, but a significant increase in weight was observed when the facility of origin is ignored. Furthermore, the observed increased weight post-diet change for females is confounded by increased rates of pregnancy (Fig. S1D). However, male marmosets not subject to that confounding variable did display a significant increase in weight.

Due to the method of fecal sample collection (see Materials and Methods), the following molecular and microbial analyses are not specific to the changes in the individual animals but rather are specific to the eight male-female pairs.

### Diet transition changes the composition of the gut microbiome

Because diet is known to have large and reproducible effects on the gut microbiome (27), we expected to see significant shifts in the gut microbiome after switching from the biscuit to the gel diet. However, despite strong clinical and phenotypic changes, we did not see significant overall community differences in either alpha (Faith’s PD Kruskal-Wallis *P* = 0.32, Shannon Kruskal-Wallis *P* = 0.52, observed features Kruskal-Wallis *P* = 0.31) or beta diversity [Fig. S2A, RPCA permutational multivariate analysis of variance (PERMANOVA), *P* > 0.8; phylo-RPCA PERMANOVA, *P* > 0.8; weighted UniFrac PERMANOVA, *P* > 0.8] when grouped by experimental phase (pre-/post-diet change).

Although we did not see significant overall effects of diet change, we did see a strong response to diet change for each unique pair (Fig. S2B, PERMANOVA *P* = 0.0001). When cohoused pair marmoset samples were examined, the expected strong effects of diet change were seen. Like in humans, the marmoset gut microbiome appears to have individualized responses to dietary changes (28). Both marmosets that had gel diet available as part of their supplemental foods (Fig. S2C, Asami and Beryl, Johnny and Rene) and those that did not (Fig. S2D, Ellen and Mac, Tammy and Bane [TB]) responded to the change in their primary diet. Pair responses even extended to differential abundance (ANCOM-BC and RPCA ranked differentials) and resulting log ratios (Fig. S2E and F). Log ratios were used instead of relative abundances since they are much more reliable and repeatable due to the compositional nature of microbiome data (29, 30). Log ratios made from differentially abundant features for Tammy/Bane at the genus (Fig. S2E) and operational genomic unit (OGU) level (Fig. S2F) did not generalize to the rest of the pairs. Even in the case of Fig. S2F, where pair Johnny/Rene had significant differences in the log ratio between pre- and post-samples just like TB, the log ratio balance was tipped in the opposite direction.

**Fig 2:**
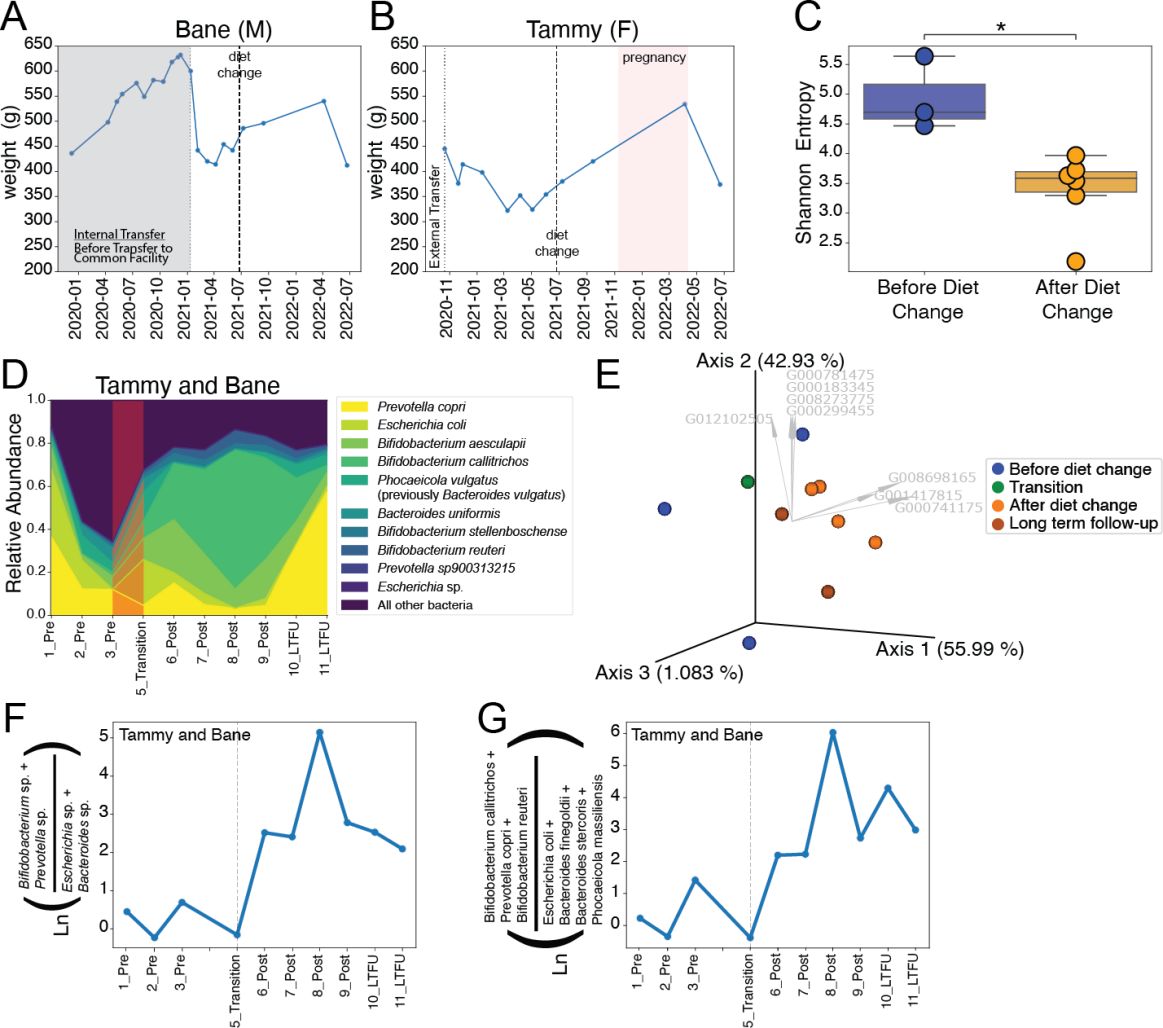
Diet change response for Tammy and Bane over time. (**A**) Lineplot of marmoset “Bane” (male) weight over time. Gray shaded region indicates weights before moved to the common breeding facility of the study. (**B**) Lineplot of marmoset “Tammy” (female) weight over time. Pink shaded region indicates pregnancy. (**C**) Shannon alpha diversity for Tammy and Bane before and after diet change. Significance was determined by Kruskal-Wallis Test. *P value < 0.05. (**D**) Relative abundance of the top 10 most prevalent bacteria post-diet change, over time. (**E**) RPCA Emperor PCA biplot colored by time point category. Arrows (gray) indicate the top eight OGU drivers of the data. (**F**) (Genus level—all OGU in each genus) Natural log (Ln) ratio of *Bifidobacterium* and *Prevotella* spp. compared to *Escherichia* and *Bacteroides* spp. for TB over time. Comparison to other marmosets can be found in Fig. S4E. (**G**) Natural log (Ln) ratio of seven OGU of interest for TB over time. Comparison to other marmosets can be found in Fig. S4F.

Since responses to diet change were unique to each pair, TB are highlighted since they had a strong response to diet change, including fertility. TB did not have detectable pregnancy prior to the diet change but did have a successful pregnancy after the diet change (Fig. 2A and B). Unfortunately, sample size for unique pairs is small and lacks enough power to find all but the largest of differences. Future studies with a larger sample size will be beneficial to show that this is consistent with the diet change. We found no significant differences between pre-diet-change and post-diet-change samples for alpha diversity metrics that assessed biodiversity (Faith’s PD Kruskal-Wallis *P* = 0.61) and the presence of unique bacteria (observed features Kruskal-Wallis *P* = 0.61), but did find significant differences in the richness and evenness of taxa present (Shannon Kruskal-Wallis *P* = 0.02) (Fig. 2C). This is supported by the observed changes in the relative abundance of the top 10 most prevalent bacteria over time (Fig. 2D). In general, no significant differences in beta diversity were seen (weighted UniFrac PERMANOVA *P* = 0.064, RPCA PERMANOVA *P* = 0.082, and phyloRPCA PERMANOVA *P* = 0.108), but samples pre- and post-diet change did appear to cluster together (Fig. 2E). Natural log ratios made from the top ranked differentially abundant bacteria for TB at the genus (Fig. 2F) and OGU level (Fig. 2G) were made and demonstrated strong changes over time. Both log ratios indicate that bacteria such as *Escherichia coli* and *Bacteroides* spp. predominate TB’s gut microbiome on the biscuit diet, whereas *Bifidobacterium* spp. and *Prevotella copri* predominate when the pair are on the gel diet. This is consistent with previous findings that increased levels of *Bifidobacterium* and *Prevotella* are important for marmoset digestion (5, 13).

We also examined the KEGG ortholog gene pathways present and found pathways associated with copper and iron (copper chaperone, iron-complex transporter, and heme-complex transporter) to be differentially regulated (Fig. S2G). Metal ions are in high demand in the gut environment and have been linked to gut dysbiosis (31).

### Metabolites correlated with the improved health of common marmosets after diet change

**Fig 3:**
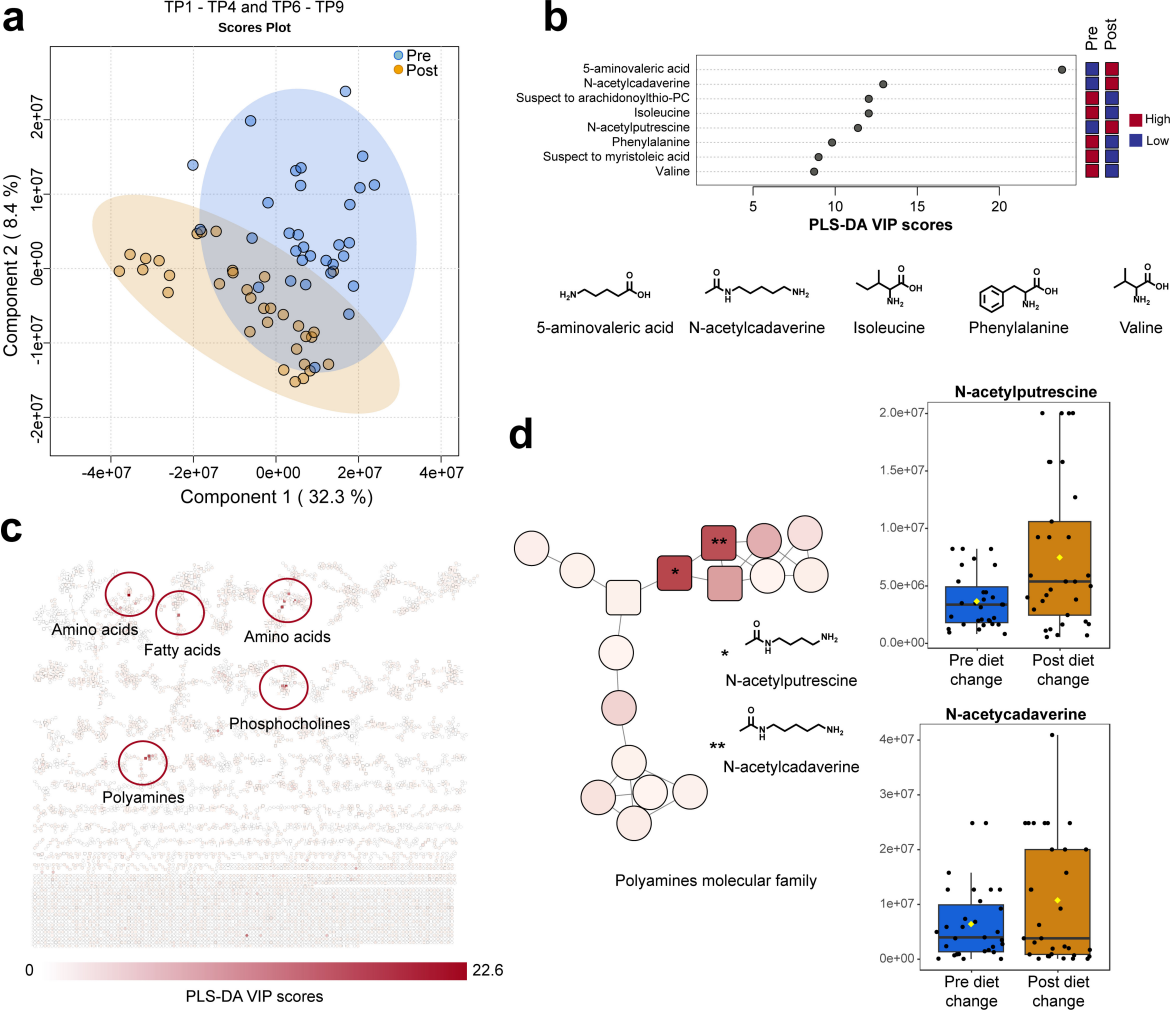
Impact of diet change on fecal metabolome. (a) PLS-DA analysis of fecal samples before (time points 1–4, Fig. 1A) and after (time points 6–9, Fig. 1A) diet change. (b) Top eight annotated molecules detected and identified using spectral libraries in GNPS. Chemical structures of representative identified molecules are shown. (c) Molecular networking highlighting molecular families, such as polyamines-related molecules, and color coded with the VIP scores from PLS-DA analysis. (d) Molecular family of polyamines, highlighting N-acetylputrescine and N-acetylcadaverine in pre- and post-diet change groups. Identification of molecules of interest was performed using spectral similarity to reference libraries in the GNPS platform.

Fecal samples, dietary samples (gel and biscuit), as well as additional supplements and treats, were assessed using untargeted LC-MS/MS. The acquired data were analyzed using molecular networking within the GNPS infrastructure (32, 33). A total of 6,094 detected molecules (from both fecal and dietary samples) with 1,183 unique features were annotated using spectral libraries resulting in a ~19% annotation rate with a 1% false discovery rate (FDR) (34–36). After the analysis of the fecal samples corresponding to time points 1–4 (pre-diet change) and 6–9 (immediate post-diet change), there were only 189 unique molecular features with significant changes [log_2_(fold change) at 0.05 FDR] (Fig. S3; Table S1) and high VIP scores from PLS-DA analysis (Fig. 3a; Table S2). There was a significant shift in fecal metabolites between pre- and post-diet transition (all metabolites, RPCA distance matrix, pre vs post, PERMANOVA *P* < 0.001). Significant shifts were seen as a group (Fig. 3a) as well as in unique pairs (Fig. S4A through J). Pairs have similar but unique responses to diet change with some pairs responding more strongly than others (Fig. S4A through J). This information was merged into the generated molecular network to facilitate the visualization of chemically related molecules. Members of the polyamine molecular family (Fig. 3b through d) were selected as features of importance by statistical analysis, indicating the significant impact of the diet change on the polyamine pathway.

### Correlational analysis of metabolomics and metagenomics indicates that there is a link between the microbes and metabolites of interest

**Fig 4:**
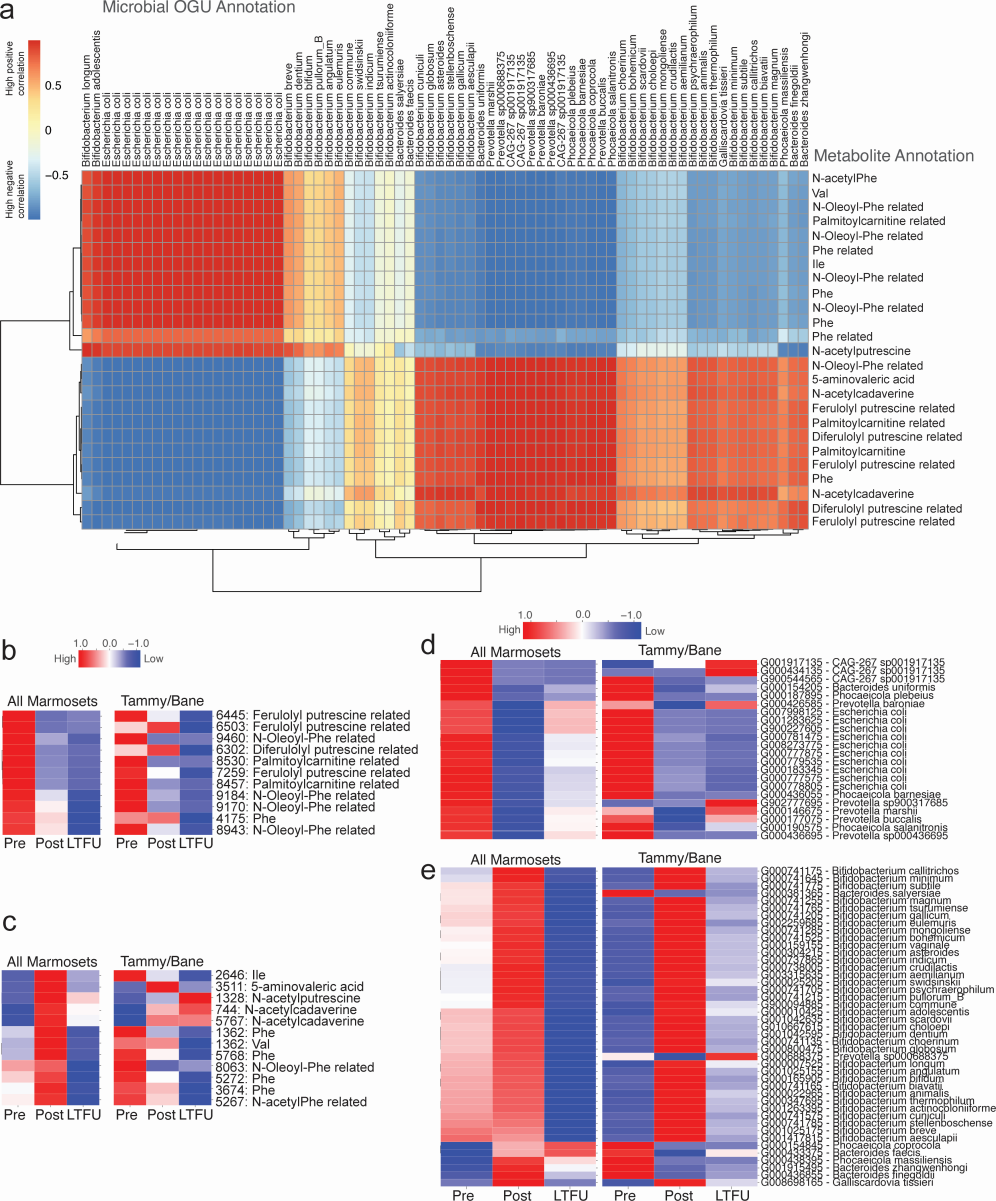
Metabolomics and metagenomics correlation for metabolites and microbes of interest. Joint-RPCA builds upon the ability to account for compositionality and sparsity using the robust center log-ratio transform but does so with a joint sample factorization across ‘omics (37, 38). (a) Joint-RPCA correlation matrix for the top differentially abundant microbes and metabolites as identified in each of the single ‘omics data sets using the full data set with all marmosets (Fig. 2 and 3). For metabolomics, if there is a direct match to a reference spectrum of a known molecule (spectral library), just the name is reported. If a spectral match is done to a related molecule (suspects library), the annotation with “related” is reported (36). (b) Clustermap of the key metabolites that were higher before diet change and lower post-diet change by mean raw absorbances grouped via the phase of experiment Pre (TP1–4), Post (TP6–9), and LTFU (TP10–11), with each row normalized by *z*-score for all marmosets (left) and the Tammy/Bane pair (right). (c) Clustermap of the key metabolites that were higher post-diet change by mean raw absorbances grouped via the phase of the experiment, with each row normalized by *z*-score for all marmosets (left) and the Tammy/Bane pair (right). (d) Clustermap same as panel b, but for the key microbes (OGU) that were higher before diet change and lower post-diet change. (e) Clustermap same as panel c, but for the key microbes (OGU) that were higher post-diet change.

Overall, feruloyl putrescines (cinnamate coumaric acid) and N-oleoyl-phenylalanine (fatty acyl amide) are higher pre-diet transition and lower post-diet and during long-term follow-up for all marmosets (Fig. 4a and b). Phenylalanine (amino acid), N-acetylphenylalanine (cyclic amino acid), valine (amino acid), 5-aminovaleric acid (omega-amino fatty acid), isoleucine (amino acid), N-acetylputrescine (polyamine), and N-acetylcadaverine (polyamine) increase immediately post-diet change but drop off during long-term follow-up for all marmosets (Fig. 4c). When we look at a unique pair, we see that those trends are not fully replicated. For Tammy and Bane, we see that N-acetylcadaverine increases post-diet change and persists at elevated levels long term. Unlike the marmosets overall, Tammy and Bane show phenylalanine and N-acetylphenylalanine start at high levels that drop off over time.

When looking at the microbes of interest, we generally found that *E. coli*, *CAG-267* species, and a few *Prevotella* species (not including *copri*) were higher during pre-diet transition and lower post-diet and during long-term follow-up for all marmosets (Fig. 4d). Most *Prevotella* species for all marmosets, including *P. copri*, were generally low during both pre- and post-dietary transition with higher levels seen during long-term follow-up. In addition, *Bifidobacterium* species, including *Bifidobacterium callitrichos*, were generally present at moderate levels before diet transition, high levels in the month immediately post-diet, and low levels during long-term follow-up (Fig. 4e). *Bacteroides faecis* and *Phocaeicola coprocola* were two species that were lowest during the pre-diet transition and gradually increased over time to their highest levels during long-term follow-up when looking at all marmosets but were very different when looking at Tammy and Bane alone. Different individual microbial responses likely contributed to the unique microbial compositions and metabolite levels seen.

## DISCUSSION

Molecular and microbial analyses of fecal samples collected during a dietary transition in a breeding colony of common marmosets confirmed that a gel diet (Mazuri Callitrichid gel diet) with an insect-based protein source is more beneficial as determined by clinically observed improvements in physiologic and reproductive outcomes compared to a biscuit diet (Lab Diet 5040) with an animal-based protein source. Since an insect-based diet may reflect the diet eaten in the wild better, it may contribute to the health improvements seen, but more research is needed.

Microbiome results indicated that *Bifidobacterium* species were generally increased after dietary transition. We know from previous research that common marmosets are colonized from infancy and throughout their lives by a community of *Bifidobacterium* species with species-specific genomic content (19). This is supported by previous literature (13) that shows significant genomic differences between strains of *Bifidobacterium* that enable the metabolism of marmoset dietary substrates, specifically carbohydrates, within the same host. Considering that *Bifidobacterium* species, in general, increased post-diet transition with the gel diet where clinical health outcomes were improved, we suspect that these microbes are involved in host-microbe interactions, including relevant biotransformation of food molecules that result in improved clinical outcomes. In our study, the marmoset-specific strain, *Bifidobacterium callitrichosis*, was increased after dietary transition. Previous literature found that *Bifidobacterium callitrichos* is more prevalent in wild forest marmosets than in captive ones (39), meaning that the gel diet with its insect-based protein source may help foster a more “normal” microbiome for this species. Of importance, this may indicate that commercially available probiotics containing *Bifidobacterium* species might be more beneficial than the slightly more common *Lactobacillus* strains. In fact, in three follow-up case reports from different buildings on the same campus, marmosets on a gel diet with chronic diarrhea that failed to resolve after multiple antibiotic therapy courses (enrofloxacin, metronidazole, and trimethoprim sulfate) and/or corticosteroid therapy (budesonide) were identified. Initial supportive therapy also included kaopectate/bismuth subsalicylate and a *Lactobacillus*-based probiotic. All three cases resolved 4–6 weeks after switching to a *Bifidobacterium*-based probiotic (Visbiome, an off-the-shelf product for humans) with no recurrence of diarrhea after 1–2 months and, therefore, was considered to be useful in diarrhea resolution. Future studies need to establish the reproducibility of the result with a larger sample size and the generality of the lack thereof in other strains. Future studies may also find it beneficial to culture bacteria, such as *Bifidobacterium* species, from fresh feces from each individual immediately after being produced for the purposes of identification and characterization of possible probiotics. Perhaps the culturing and administration of a marmoset-specific *Bifidobacterium* strain, such as *Bifidobacterium callitrichosis*, could also be pursued. To our knowledge, there are no marmoset host-derived *Bifidobacterium callitrichosis* strains on the market, which may be an even better choice as a probiotic. Further research is required to examine this concept further.

In addition, *Prevotella copri* was shown to be increased in several unique pairs after dietary transition when clinical improvement was seen. *Prevotella copri* is known to help digest carbohydrates and increased prevalence has been associated with IBD and other chronic gastrointestinal inflammatory states (12, 40). While we found significantly higher amounts of many *Prevotella* species early in the study when the marmosets were not doing well, *Prevotella copri* was not among them. It is possible that since 16S is not accurate for species-level annotations (41), previous reports that *Prevotella copri* was associated with disease actually referred to another species of *Prevotella* or that the *Prevotella copri* strains present in these marmosets do not have host detrimental genes present. Therefore, future work investigating *Prevotella* strains is suggested to determine whether there are certain strains of *Prevotella* and the functions of those strains that correspond to health and disease in captive laboratory marmosets.

Of the top 36 differentially present microbial genes determined from our shotgun metagenomics data, seven involve metal ion transport (iron, copper, or nickel). These metal ions, considered to be trace elements, can play critical roles in meeting the host’s demands to maintain normal biological, cellular, and metabolic functions. Previous literature has described the concentrations of iron uptake and metabolism in *Bifidobacterium*, which contributes to its beneficial effects (42). *Bifidobacterium* has also been shown to have constant urease activity, in which nickel cations are necessary for proper functioning of the gut microbiota. In general, the gut microbiome can compete with the host to obtain or sequester these trace elements to prevent bacterial access, which can lead to dysbiosis (31). Trace elements may be required from the diet for normal biological processes, but when in excess, they are excreted from the gut, as has been shown in other species like pigs or poultry (43). Excess or inadequate amounts of trace metal elements in the host can change the intestinal microbial community structure and function (44). The gut is key to maintaining the homeostasis of trace minerals, with modulation and excretion into the gut. Thus, there is a dynamic role between the host, microbiome, and trace elements that help shape the gut microbiome. Trace elements have also been shown to play a role in the commonly used probiotics, *Bifidobacterium* and *Lactobacillus*, to help balance the gut microbiota and, subsequently, to aid in maintaining proper body functioning (31, 42). Therefore, the trace elements found here and their role in the changes to the bacteria present post-dietary change correspond to our findings that the gel diet improved the gut health of our common marmosets. Moreover, the inconsistent group changes and structures in the microbiome do not necessarily reflect the functional importance of the trace elements and genes of the microbes present within the common marmoset microbiome. These results mimic the findings seen in the human microbiome, where there tends to be unique microbial compositions but commonalities in function (45, 46). This further highlights the value of common marmosets as a model for human studies since they are less inbred and have more immunological and microbial diversity than common laboratory mice, which are genetically homozygous and have minimal variability.

Untargeted metabolomics results highlighted amino acids, fatty acids, phosphocholines, and polyamines as being key changes after dietary transition, which is consistent with previous metabolomics studies performed on plasma from common marmosets (24, 30). From the highlighted metabolites (Fig. 2B), two were polyamines and four were amino acids. These molecular families are important in protein metabolism, cellular growth and development, immune responses, and gene expressions. Differences in their pathways are important to understand different disease mechanisms, such as aging and longevity, cardiovascular and gastrointestinal diseases, as well as neuromodulatory diseases (24, 30, 31). Notably, polyamines present in the lumen of the gastrointestinal tract may have different origins, and their levels depend on their uptake from the gut microbiota or from different dietary conditions. Polyamines, which contribute to the maintenance of intestinal homeostasis, can be beneficial to the health of the gut, though at too high concentrations, they can be harmful (47, 48). They are tightly controlled and their quantities can be altered, which can influence a number of pathological conditions, such as cancer, obesity, and diabetes (49–51). Specifically, the polyamines, putrescine and cadaverine, have shown cytotoxicity in intestinal cells, and accumulation of these toxic compounds can be detrimental to the gut (47). Thus, increased excretion of these molecules, rather than potential interaction or reabsorption by intestinal cells, may have a positive outcome and potential benefits. This is consistent with what we found here, where the increased levels of the polyamines, cadaverine and putrescine, in the fecal samples post-diet change indicate that they were excreted from the body, such that they were metabolized in the gut to levels that prove to be beneficial to the animal. Previous studies have used plasma to analyze fatty acids (24) or have been observational, focusing on short-chain fatty acids (SCFAs) in cohabitating animals rather than associated with diet change (52). Our study provides the first public fecal metabolome data set associated with a diet change. This untargeted metabolomics approach enabled us the detection of thousands of non-volatile molecules; therefore, SCFAs were not considered. Although there are no reports on the impact of diet on the metabolism of SCFAs, it would be of interest to investigate this range of molecules in future studies. Taken together, the metabolome showed significant compositional shifts that are associated with improved health of the marmosets after dietary transition. Overall, molecules positively associated with health and clinical improvement, specifically, the polyamines, which significantly increased post-diet change, should be considered for follow-up studies investigating their potential beneficial role.

While we are unable to conclusively determine whether there were additional external or environmental factors that could have contributed to the decrease in weight and lack of reproduction, we are confident that diet was the primary factor contributing to a decline in health. There were no other noted significant events or changes to their environment that could have led to clinical decline. After the quarantine period and co-housing, diet was the only major housing component that changed for the colony during the critical time window. However, based on experience, we would have expected the animals to have acclimated to their new environment and partners within 2–3 months. We also considered that pregnancies, whether successful or not, could have influenced our interpretation of the weight gains and that compatibility, or lack thereof, between the male-female pairs could have influenced the breeding potentials. However, our overall assessment over the course of the year analyzing the colony leads us to believe that there was a significant improvement in weights with the gel diet compared to the biscuit diet, regardless of pregnancy status, due to the significant weight changes in the male marmosets.

Another consideration to be made is that water intake of the individual animals was not measured. In the future, we plan to note whether the water intake remained the same or increased when fed a biscuit versus a gel diet. If water content appears to be a critical factor, we also plan to examine how adding moisture such as Tang juice or apple juice to a biscuit diet might make a difference. In the few cases we observed where additional moisture was added, marmosets did not appear to consume more pellets nor did clinical signs appear to resolve. This anecdotal evidence that moisture content alone is not the key factor needs data from a well-designed study to confirm. Additionally, while only one specific biscuit diet (Lab Diet 5040) and gel diet (Mazuri Callitrichid gel diet) were analyzed and compared in our study, it would be worthwhile to analyze the nutritional compositions of other forms of biscuit and gel diets, as well as the fecal microbial and metabolic profiles of marmosets fed these diets. Moreover, analyses for other forms of diets, such as a canned diet like Zupreem, should be performed to determine whether there is a hydration component to the diets fed that led to clinical improvements.

Since additional food supplements are typical of most university institutions, there can be a wide variety of enrichments provided to a colony, including live or freeze-dried feed (e.g., crickets and mealworms) or treats (e.g., yogurt, trail mix, and marshmallows). While additional food supplements were provided to our colony as a source of enrichment, such as acacia gum, mealworms, crickets, and hard-boiled eggs, these were held constant pre- and post-primary diet change, and we cannot determine how changes in these components may have affected health parameters. We acknowledge and emphasize the importance of the exudivorous nature of common marmosets and the need to have tree gum and insects included in their captive diet to replicate their wild diet. Since the focus of our paper was on the main diet in captivity, we highly recommend that further work be done to study how changes in these food enrichments, especially tree exudates/gum, affect health. This is warranted as they encourage normal behavior and can help us more closely mimic the natural diets of marmosets.

While data were not actively collected after the year of monitoring and analyzing the colony, several of the co-authors are laboratory animal veterinarians and can confirm that the breeding colony continues to thrive, and the breeding outcomes and success have continued to the present day. Our study is the first to use both shotgun metagenomics and LC-MS/MS untargeted metabolomics to identify features that were beneficial to the gut when fed a gel diet like Mazuri Callitrichid gel diet, compared to a biscuit diet like Lab Diet 5040. However, since our findings were more pair-specific rather than consistent across the entire colony, further studies are needed to decipher whether these molecules and metabolites are beneficial for individual marmosets in a larger population and whether there are age and sex differences. Age and sex were not considered a factor when determining the differences in molecular and microbial profiles from food and feces due to the pooled collection method but can and should be considered in future studies. We also acknowledge that the cost and personnel time required to prepare and maintain a gel diet is more than that for a biscuit diet for a colony of marmosets. While those factors often heavily influence choosing the biscuit over the gel diet, our study provides evidence of other factors that should also be heavily weighted in this decision. Many of the molecules and microbes identified in our study have been found in the gut microbial composition of humans, which further enhances our knowledge of translatability between common marmosets and human beings. This study also suggests that the marmoset may serve as a useful model to study gut microbial diversity and proposes that the microbiome should be considered as a biological variability, as it is fundamental to the host phenotype, which can be influenced by the source, the genetic makeup, the housing conditions, and, in particular, their diet. Therefore, differences in the microbiome and its changes depending on the diet fed and other environmental factors can impact the health of the animal and the data being produced and should be documented in experiments. In conclusion, diet can clearly affect both the microbiome and metabolome in ways that impact the health of the animal and, subsequently, the experimental outcomes.

## MATERIALS AND METHODS

### Animal and dietary information

A total of 16 adult common marmosets (eight males and eight females) of mixed age and body mass were housed in male-female pairs at an off-campus AAALAC-accredited facility at the University of California, San Diego, in accordance with the Guide for Care and Use of Laboratory Animals (“The Guide”). Of the 16 marmosets, 9 arrived from two external institutions, and the remaining 7 of 16 were introduced from the internal main-campus colony at UCSD, with the intent to create a breeding colony and increase genetic diversity. Arrival and pairing dates varied. All animals received general health exams, including hematological and chemistry blood work, fecal flotation for ova and parasites, and fecal culture for *Shigella*, *Yersinia*, *Campylobacter*, and *Salmonella*. The animals also received intradermal tuberculin tests, which were checked daily for 72 hours. Blood tests performed were overall unremarkable, fecal floats and cultures were negative for parasites and bacteria, and Tb tests were noted to be negative. All animals were reported to be healthy with good body condition and hydration status. All marmosets were fed Lab Diet 5040 (Purina, Wayne County, IN, USA) after arrival, which is a commercially processed biscuit diet, in addition to supplementary foods and treats. Lab Diet 5040 is a New World primate diet that is 20% protein (animal-based), 9% fat, and 6% fiber. Deterioration of health was observed in the colony over time. After 8 months in the facility, marmosets were transitioned off the Lab Diet 5040 and on to the Mazuri Callitrichid gel diet (Purina, Arden Hills, MN, USA), which was prepared in-house. Mazuri Callitrichid gel diet is 20% protein (insect-based), 7% fat, and 4% fiber. They continued to be fed the same supplementary foods and treats as before. Supplementary foods and treats included marshmallows, yogurt drops, fresh fruits, vegetables, mealworms, crickets, hard-boiled eggs, arabic gum, Ensure, and yogurt. Weights and reproductive outcomes were monitored and recorded over the course of the experiment.

#### Sample collection

Fecal samples were collected from cage liners from the eight pairs of marmosets at approximately the same time each morning (within a 4-hour window—8 a.m. to noon; ZT2-6), placed in individual sterile 1.7 mL Eppendorf tubes, and frozen at −80°C until analysis. Since samples were collected from cage liners, we acknowledge that it is not possible to differentiate samples per individual. Additionally, the feces may have residual environmental and/or urinary microbes and molecules from being on the cage floor. These fecal samples were collected at four time points prior to the diet change (twice 3 weeks before, once 2 weeks, and once 1 week before the diet change; biscuit diet only), once during the transition week (gel and biscuit), and at four time points post-diet change (twice in the first week, once in the second week, and once 1 month post-diet change; gel diet only). Fecal samples were also collected approximately 10 and 13 months post-diet change (gel diet only). These fecal samples were divided for both metagenomic and metabolic analyses. Samples of both the biscuit and gel diet, as well as a variety of supplementary foods and treats, were also collected in sterile 1.7 mL Eppendorf tubes and frozen at −80°C until metabolomic analysis.

### Untargeted tandem mass spectrometry-based metabolomics

#### Sample extraction

Extraction solvent (methanol:water, 50:50, vol/vol) was prepared and stored at 4°C the day before the extraction procedure. Samples were extracted by adding 800 µL of cold solvent (4°C methanol:water, 50:50, vol/vol) per 20 mg of stool (sample to solvent ratio 1:40) containing a clean steel bead. Samples were homogenized at 25 Hz for 5 min using a tissue lyzer, incubated at 4°C for 30 min, and centrifuged at max speed (>15,000 × *g*) for 10 min at 4°C (Sorvall Legend RT, Marshall Scientific, Hampton, NH, USA). A volume of 440 µL of each sample was transferred to a 96-shallow-well plate. Samples were dried down using a Centrifugal Vacuum Concentrator, Centrivap (Labconco, Kansas City, MO, USA) and stored at −80°C until LC-MS/MS analysis.

#### LC-MS/MS acquisition

Sample extracts were dissolved in 200 µL of 50% methanol:water containing 1 µM sulfadimethoxine as an internal standard for LC-MS monitoring. Untargeted LC-MS/MS acquisition was performed on a Vanquish Ultrahigh Performance Liquid Chromatography system coupled to a Q-Exactive Hybrid Quadrupole-Orbitrap (Thermo Fisher Scientific, Bremen, Germany). Chromatographic separation was performed on a Kinetex 2.6 µm 100 Å pore size Polar C18 reversed-phase UHPLC column 150 × 2.1 mm (Phenomenex, Torrance, CA, USA) with a constant flow rate of 0.5 mL/min. The following solvents were used during the LC-MS/MS acquisition: water with 0.1% formic acid (vol/vol), Optima LC/MS Grade, Thermo Scientific (solvent A) and acetonitrile with 0.1% formic acid (vol/vol), Optima LC/MS Grade, Thermo Scientific (solvent B). After injection of 5 µL of sample into the LC system and eluted with an isocratic gradient of 5% B from 0 to 1 min and linear gradient from 5% to 25% B (1–5 min), 25% to 99% B (5–7 min), 99% B (7–8 min), 99% to 5% B (8–8.5 min), and 5% B (8.5–10 min). Data-dependent acquisition (DDA) mode was used for the acquisition of tandem MS (MS/MS) with a default charge state of 1. Full MS was acquired using one microscan at a resolution (R) of 35,000 at 200 *m/z*, automatic gain control (AGC) target 5e5, maximum injection time of 100 ms, scan range of 80–1,200 *m/z,* and data acquired in profile mode. DDA of MS/MS was acquired using one microscan at a resolution (R) of 17,500 at 200 *m/z*, AGC target of 5e5, top five ions selected for MS/MS with isolation window of 1.0 *m/z* with a scan range of 200–2,000 *m/z*, fixed first mass of 50 *m/z,* and stepped normalized collision energy of 20, 25, and 30 eV, minimum AGC target of 2.50e4, intensity threshold of 2.5e5, apex trigger from 2 to 15 s, all multiple charges included, isotopes were excluded, and a dynamic exclusion window of 5 s. Analytical blanks and a mixture of sulfamethazine, sulfamethizole, sulfachloropyridazine, sulfadimethoxine, amitriptyline, and coumarin-314 (10 µM) were injected after every 48 samples as quality control for monitoring instrument (LC-MS) performance.

#### Pre-processing and molecular networking

Pre-processing was performed using MZmine3 (53) to generate input tables for feature-based molecular networking. A molecular network was created using the online workflow (https://ccms-ucsd.github.io/GNPSDocumentation/) on the GNPS website (35) (http://gnps.ucsd.edu). The data were filtered by removing all MS/MS fragment ions within ±17 Da of the precursor *m/z*. MS/MS spectra were window filtered by choosing only the top six fragment ions in the ±50-Da window throughout the spectrum. The precursor ion mass tolerance was set to 0.02 Da with an MS/MS fragment ion tolerance of 0.02 Da. A network was then created, where edges were filtered to have a cosine score above 0.65 and more than four matched peaks. Furthermore, edges between two nodes were kept in the network if and only if each of the nodes appeared in each other’s respective top 10 most similar nodes. Finally, the maximum size of a molecular family was set to 100, and the lowest-scoring edges were removed from molecular families until the molecular family size was below this threshold. The spectra in the network were then searched against GNPS’ spectral libraries (35, 36). All matches kept between network spectra and library spectra were required to have a score above 0.83 and at least four matched peaks to achieve a 1% FDR. This method resulted in 1,183 annotated metabolites (20% of all detected metabolites). The molecular networking analysis can be accessed through the link: https://gnps.ucsd.edu/ProteoSAFe/status.jsp?task=2af71670bbd84788bfa63782cefe6e3f.

Additionally, molecular networking using bile acid libraries was performed and can be accessed through the following link: https://gnps.ucsd.edu/ProteoSAFe/status.jsp?task=7165f4739598461db161973709a856c2.

#### Statistical analysis of features detected by LC-MS/MS

Statistical analyses were performed using the MetaboAnalyst 5.0 platform (54). The peak intensity table obtained after preprocessing with MZmine3 software was filtered to only consider fecal samples from paired marmosets and then uploaded to MetaboAnalyst. The uploaded data file contains 80 (samples) by 6,094 [peaks (*m*/*z*/rt)] data matrix. The data were organized into two groups corresponding to PRE- and POST-diet change for sample collection and contained all time points (TP1–11, Fig. 1) for only paired marmosets analyzed in this study. Standard deviation was used to filter variables with nearly constant values. After filtering, the data containing 80 samples and 3,392 [peaks (*m*/*z*/rt)] were normalized by using quantile normalization. The normalized table used for downstream analysis can be found as Table S1 (data normalized TP1–11). The data were additionally filtered to consider only the time points TP1–4 and TP6–9 and organized into two groups, PRE and POST, corresponding to the pre- and post-diet change (Table S2, TP1–4 and TP6–9 data normalized). This file contains 64 (samples) by 2,007 [peaks (*m*/*z*/rt)] data matrix.

### Shotgun metagenomics

#### DNA extraction

Fecal samples were transferred (0.03–0.04 g) into 1 mL Matrix Tubes (ThermoFisher, Waltham, MA, USA) before gDNA extraction. Fecal samples were extracted for microbiome sequencing using reagents from the MagMAX Microbiome Ultra Nucleic Acid Isolation Kit (ThermoFisher Scientific, Waltham, MA, USA), as updated in reference 55. The protocol was adapted in order to perform the lysis and bead beating extraction steps in the 1 mL Matrix Tube sample collection devices, eliminating the sample-to-bead plate transfer step and minimizing potential cross-contamination from vortexing during bead beating . After bead beating, samples were transferred to KingFisher plates (ThermoFisher, Waltham, MA, USA), and the remaining gDNA extraction steps were performed as outlined in reference 55.

#### Metagenomics library preparation

Extracted gDNA was quantified using the Quant-iT PicoGreen dsDNA Assay Kit (Invitrogen, Waltham, MA, USA) and normalized to 5 ng in 3.5 µL sterile water for library preparation, which was performed using a miniaturized adaptation of the KAPA HyperPlus Library Kit (Roche, Basel, Switzerland), as outlined in reference 56.

#### iSeq normalized pooling

The library was quantified via PicoGreen Assay (Invitrogen), and all samples were equal volume pooled, PCR cleaned (Qiagen), and size selected from 300 to 700 bp using a Pippin HT (Sage Sciences). QC was run on an Agilent 4000 Tapestation (Agilent, Santa Clara, CA, USA) to confirm expected library sizes after PCR cleanup and size selection. The equal volume pool was sequenced on an iSeq100 (Illumina, San Diego, CA, USA). Utilizing the sample concentration and read counts per sample obtained from the iSeq 100 run, a normalized pooling value was calculated for each sample to optimize pooling efficiency to obtain more even read counts per sample during NovaSeq sequencing (57). After re-pooling the library with the iSeq normalized pool values, samples were PCR cleaned, size selected (300–700 bp), and QC was performed using an Agilent tapestation.

#### Sequencing and post-sequencing processing

The PCR-cleaned, size-selected, iSeq-normalized pool was sequenced on a NovaSeq 6000 (Illumina, San Diego, CA, USA) at the Institute for Genomic Medicine at the University of California, San Diego with an S4 flow cell and 2 × 150 bp chemistry. Raw sequence reads (BCL files) were demultiplexed to per sample FASTQ and quality filtered (58). Adapter trimming was performed by fastp (59), and human filtering to ensure compliance with database regulations was performed via minimap2 (60) by alignment to two databases: one containing the human reference genome GRCh38 and PhiX, and the second containing the human reference genome CHM13. The resulting FASTQ were uploaded into Qiita (61) (study ID #14577) and processed with default shotgun metagenomic parameters. Using the graphical interface, the default shotgun metagenomic analysis pipeline was performed using Woltka (version 0.1.4) (62). In brief, direct genome alignments were made against the “Web of Life” database (release 2) (63), which contains 10,575 microbial genomes of bacteria and archaea. The sequence alignment is performed using a bowtie2 aligner (64) and by mapping sequencing data to microbial reference genomes. Reads mapped to a microbial reference genome are counted as hits such that the resultant feature table comprises samples (rows) by microbial genome IDs (columns) and concomitant abundances. If a sequence maps to multiple genomes by Bowtie2 (up to 16), each genome is counted 1/*k* times, where *k* is the number of genomes to which a sequence is mapped. Microbial genome IDs are considered operational genomic units and provide a shotgun metagenomic equivalent to amplicon sequence variants in 16S rRNA amplicon sequencing data (62). The OGU frequencies were then summed after the entire alignment was processed and rounded to the nearest even integer, thereby making the sum of OGU frequencies per sample nearly equal (considering rounding) to the number of aligned sequences in the data set. The resultant count matrix is saved as a biom format table (65).

We attempted to identify individual marmosets but were unsuccessful, possibly due to mutual grooming behaviors that would have led to the presence of both individuals DNA in the digestive tract. We first tried to identify the host sex using the y-chromosome (NCBI NC_048406.1) via BWA-MEM and bowtie2 (66, 67). Further investigation revealed that the y-chromosome partially aligns with the x-chromosome (NCBI NC_048405.1), which may be why reads are mapped indiscriminately. We also attempted to identify the host sex by looking at alignments to the sry (NCBI NC_071465.1) and zfy genes (NCBI AY220126.1) via BWA-MEM and BWA-FastMap (68). Unfortunately, this approach was also unsuccessful in providing a definitive sex determination of samples. An additional attempt at individual identification was made by attempting alignments to several different marmoset mitochondria or mitochondrial D-loop segments (NCBI NC_025586.1, AB525908.1, AB572419.1, U86526.1, U88840.1, and KJ020024.1) via BWA-MEM and bowtie2, which also failed to align sufficient reads for the analysis for all samples (69).

#### Bioinformatic microbiome analysis

The biom file generated by the Qiita pipeline was subsequently downloaded and converted to a qiime2 (70) (v2023.2) artifact. Due to the overpooling of blanks during the iSeq normalization step, samples with either low starting DNA concentration (including blanks) or poor amplification with less than 3,000 counts/pooled volume were dropped from further analysis. The resulting table contained 55,995,612 reads for 82 samples with an average of 682,873 reads per sample. The feature table was rarified to a depth of 167,000 reads/sample to control for sequencing effort before performing standard alpha (Faith’s phylogenetic diversity [71], Shannon index [72], observed features [73]) and beta diversity (weighted UniFrac [74]) metrics. For log-based diversity metrics (Aitchison [75]; RPCA, phyloRPCA, and CTF [38]), an unrarified table was used for calculations and downstream analyses. Beta diversity differences were determined by permutational multivariate analysis of variance with a multiple-testing correction and an alpha level of 0.05 (76). Clustermaps, hierarchically clustered heatmaps based on Euclidean distances, were used to display some of the data (77). Data were visualized using custom Python scripts (the Python code is available at https://github.com/knightlab-analyses/marmoset-diet-change).

#### Combined microbiome and metabolome analysis

We used Joint-RPCA to look at connections between the microbiome and metabolome data (37, 38). In brief, the microbiome and metabolome raw feature tables were used as input and were transformed through the robust-centered-log-ratio transformation (robust-clr). Robust-clr handles the sparsity often found in microbiome and metabolome data well. The raw observed values were only computed on the non-zero entries and then averaged and optimized. A shared matrix was estimated across the shared samples of all input matrices. To ensure consistency, the estimated shared matrix and the matrix of shared eigenvalues across all input matrices were recalculated at each iteration. Minimization was performed across iterations by gradient descent. Cross-validation of the reconstruction was performed in order to prevent overfitting of the joint factorization. Correlations of features across the two input matrices were calculated from the final estimated matrices.

## Data Availability

Microbiome data are publicly available in Qiita under study ID #14577 (https://qiita.ucsd.edu/public/?study_id=14577). Raw sequencing data have been deposited to EBI/ENA, study PRJEB64278 (ERP149415). The metabolome data set generated for this study has been deposited online in the public repository Mass Spectrometry Interactive Virtual Environment (MassIVE) (https://massive.ucsd.edu/), MassIVE ID# MSV000090940. Code is available at https://github.com/knightlab-analyses/marmoset-diet-change.

## SUPPLEMENTAL MATERIAL

Supplemental Material msystems.00108-24-s0001.docx Supplemental figures and supplemental table legends.

Table S1 msystems.00108-24-s0002.csv Metabolomics.

Table S2 msystems.00108-24-s0003.csv Metabolomics.

ASM does not own the copyrights to Supplemental Material that may be linked to, or accessed through, an article. The authors have granted ASM a non-exclusive, world-wide license to publish the Supplemental Material files. Please contact the corresponding author directly for reuse.
