# Correction for Tang-Wing et al., “Impact of diet change on the gut microbiome of common marmosets (*Callithrix jacchus*)”

**DOI:** 10.1128/msystems.00327-25

**Published:** 2025-06-03

**Authors:** Cassandra Tang-Wing, Ipsita Mohanty, MacKenzie Bryant, Katherine Makowski, Daira Melendez, Pieter C. Dorrestein, Rob Knight, Andrés Mauricio Caraballo-Rodríguez, Celeste Allaband, Keith Jenné

## AUTHOR CORRECTION

Volume 9, no. 8, e00108-24, 2024, https://doi.org/10.1128/msystems.00108-24. It was brought to our attention that there was a misunderstanding regarding the gel diet contents: its protein source was not insect based. Thus, changes are needed as detailed below. These corrections do not change the conclusions of the paper.

Page 8, Discussion, first paragraph, first sentence: "with an insect-based protein source” should read “with xanthum gum.”

Page 8, Discussion, first paragraph, second sentence: “an insect-based diet” should read “the gel diet."

Page 8, Discussion, second paragraph, sixth sentence: “the gel diet with its insect-based protein source” should read " the gel diet.”

Page 13, Materials and Methods, first paragraph, fourth sentence from end: “20% protein (insect-based)” should read “20% protein.”

