# Supplementary material for: Impact of diet change on the gut microbiome of common marmosets (Callithrix jacchus)
Source: mSystems. 2024 Jul 8;9(8):e00108-24. doi: 10.1128/msystems.00108-24 (PMC11334461; doi:10.1128/msystems.00108-24)
Supplement: Supplemental Material — Supplemental figures and supplemental table legends. [file msystems.00108-24-s0001.docx]

## SUPPLEMENTAL MATERIAL

**
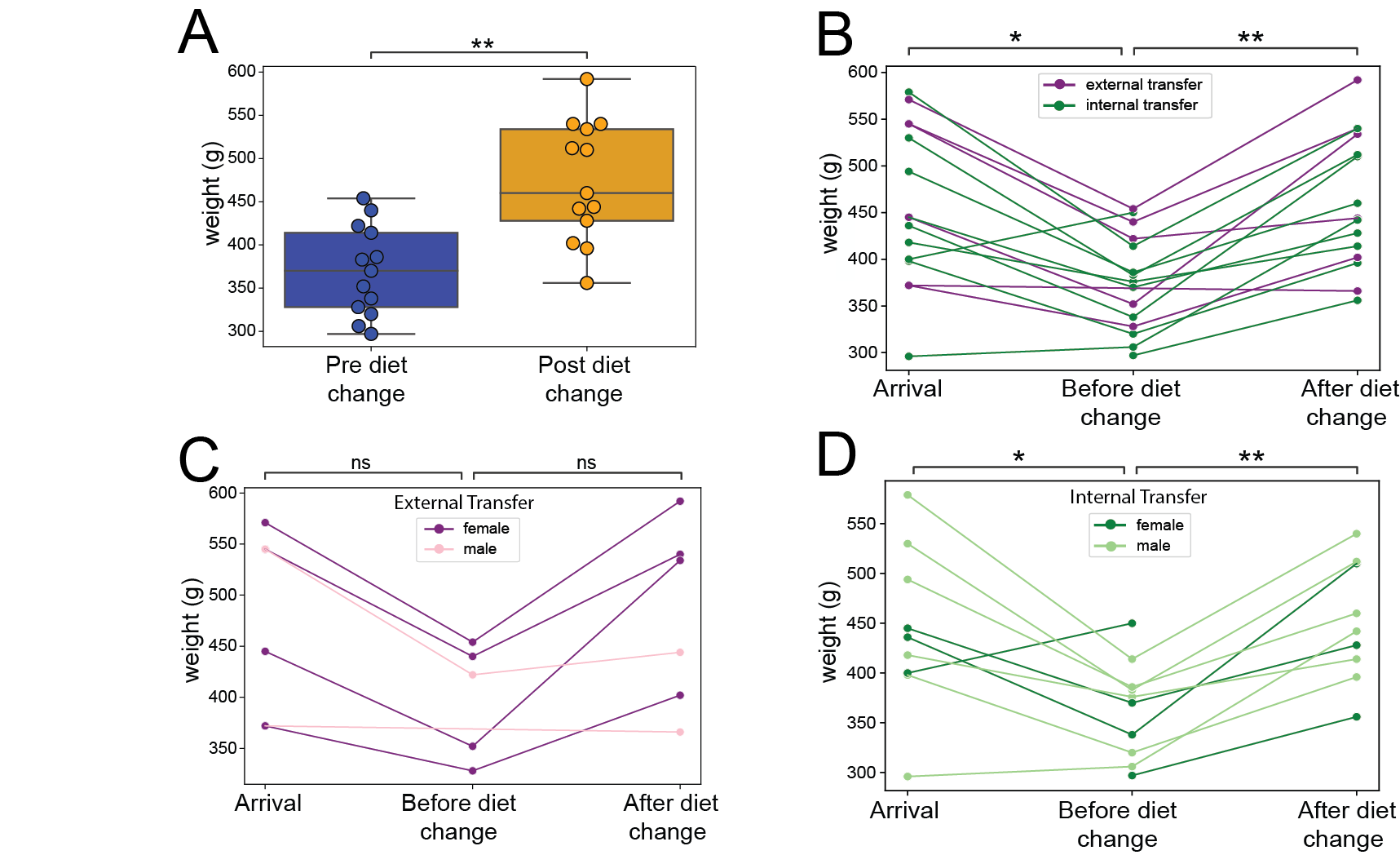
**

**Supplemental Figure 1. Additional Weight Information**

**A**) Spaghetti plot of marmoset weights over time, from arrival (October 2020), 6 months after introduction and acclimation (April 2021; fed biscuit diet), and one year later (April 2022; fed gel diet). Colored by origin of marmoset: purple for any external facility transfer (n=6), green for an internal transfer from other facilities on the same campus (n=10). **B**) Boxplot with swarmplot of marmoset weights, an indirect measurement of overall health, starting 6 months after introduction and acclimation (fed biscuit diet) and then compared to overall weights one year later (fed gel diet), separated by sex**. C**) Spaghetti plot of external transfer marmoset weights over time. There were 3 separate facilities from across the country that marmosets were transferred from. **D**) Spaghetti plot of internal transfer marmoset weights over time. One marmoset was not weighed in April 2022. Significance determined by Mann-Whitney-Wilcoxon test two-sided with Bonferroni correction. Notation: ***: p value <0.001, **: p value <0.01, *: p value <0.05.

*
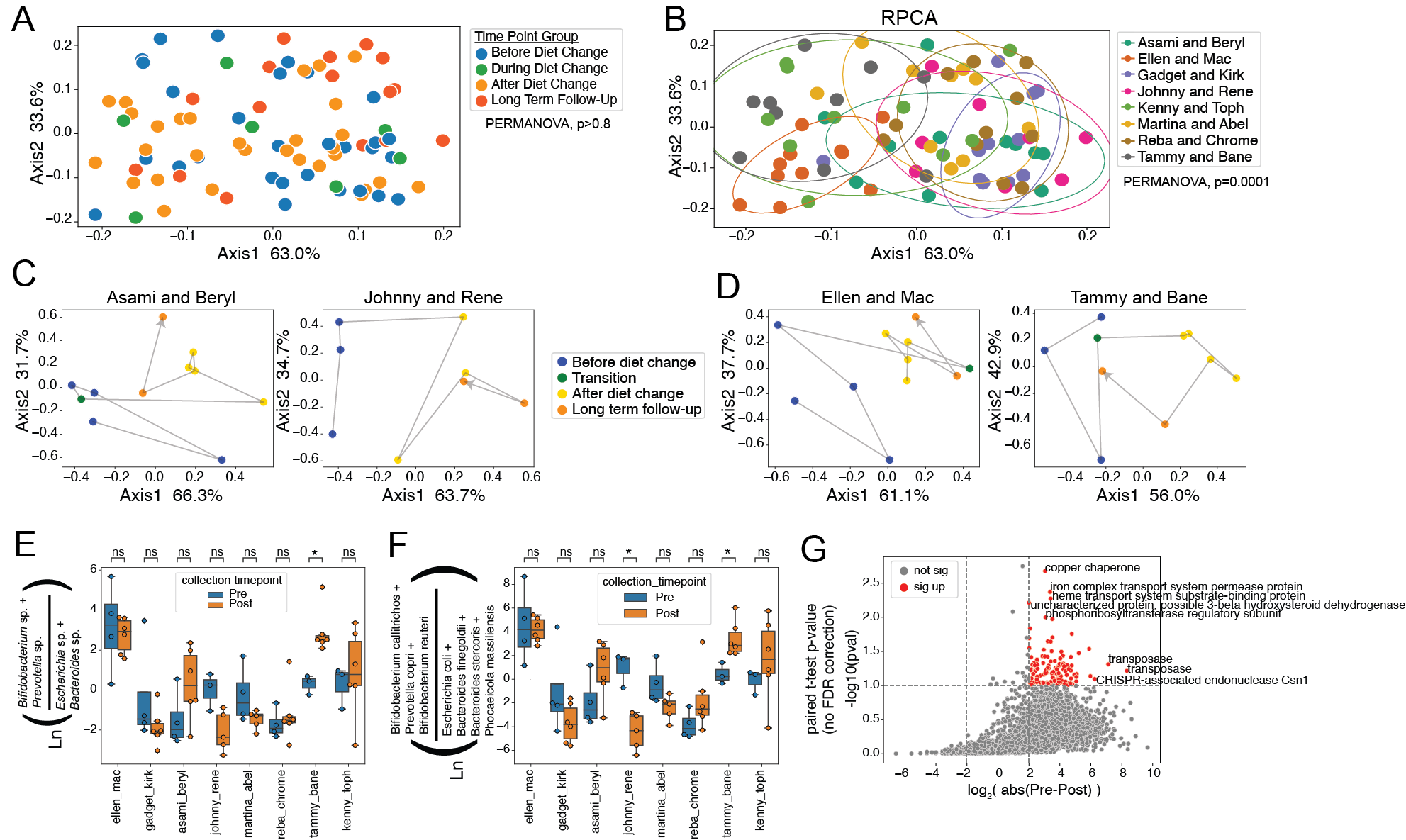
*

**Supplemental Figure 2. Diet change resulted in pair-specific microbiome responses.** **A**. RPCA PCoA plot colored by time point grouping: blue is before diet change, orange is after diet change. **B**. RPCA PCoA plot colored by marmoset pair, ellipses represent 80% confidence intervals around points corresponding to each marmoset pair. Individual Pair RPCA PCoA plot examples: **C**. Asami/Beryl, Johnny/Rene both were offered gel diets prior to full diet transition, yet still showed strong, unique responses to diet change. We were unable to get a transition sample for JR. **D**. Ellen/Mac, Tammy /Bane [TB] both showed a strong individualized effect of diet change on the microbiome. For Both C and D, a line is drawn in timepoint order and ending in an arrow. **E**. Natural log (Ln) ratio of *Bifidobacterium* and *Prevotella* sp. Compared to *Escherichia* and *Bacteroides* sp. by marmoset pair and grouped timepoints. Transition time point excluded for this analysis. **F.** Natural log (Ln) ratio of 7 species of interest by marmoset pair and grouped timepoints. Transition time point excluded for this analysis. **G.** Volcano plot using -log_10_(paired t-test p-value) by log_2_ absolute value difference between Pre and Post values. The top items with high p-value and high log fold changes are annotated. Significance determined by Mann-Whitney-Wilcoxon test two-sided with Bonferroni correction. Notation: ***: p value <0.001, **: p value <0.01, *: p value <0.05.


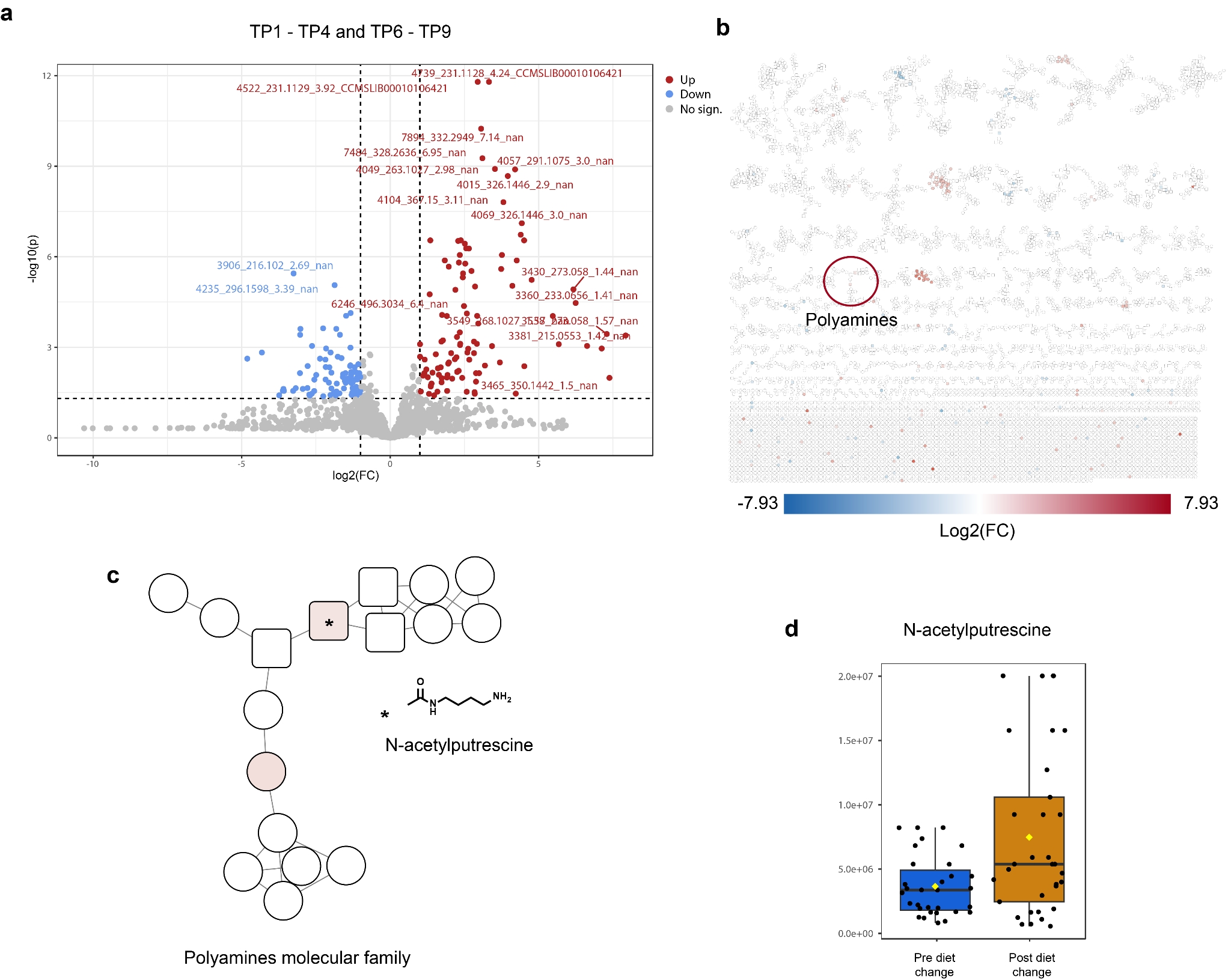


**Supplemental Figure 3**. Significant features observed before and after diet transition. (**a**) Volcano plot comparing time points 1-4 (pre-diet transition) and time points 6-9 (post-diet transition). (**b**) Molecular network mapping log2(FC) values from Volcano plot in panel a. (**c**) Molecular family of polyamines. The identification of N-acetylputrescine is based on spectral similarity to GNPS libraries (cosine 0.89). (**d**) Boxplot of detected feature identified as N-acetylputrescine in fecal samples from time points 1-4 (pre-diet change) and time points 6-9 (post-diet transition).


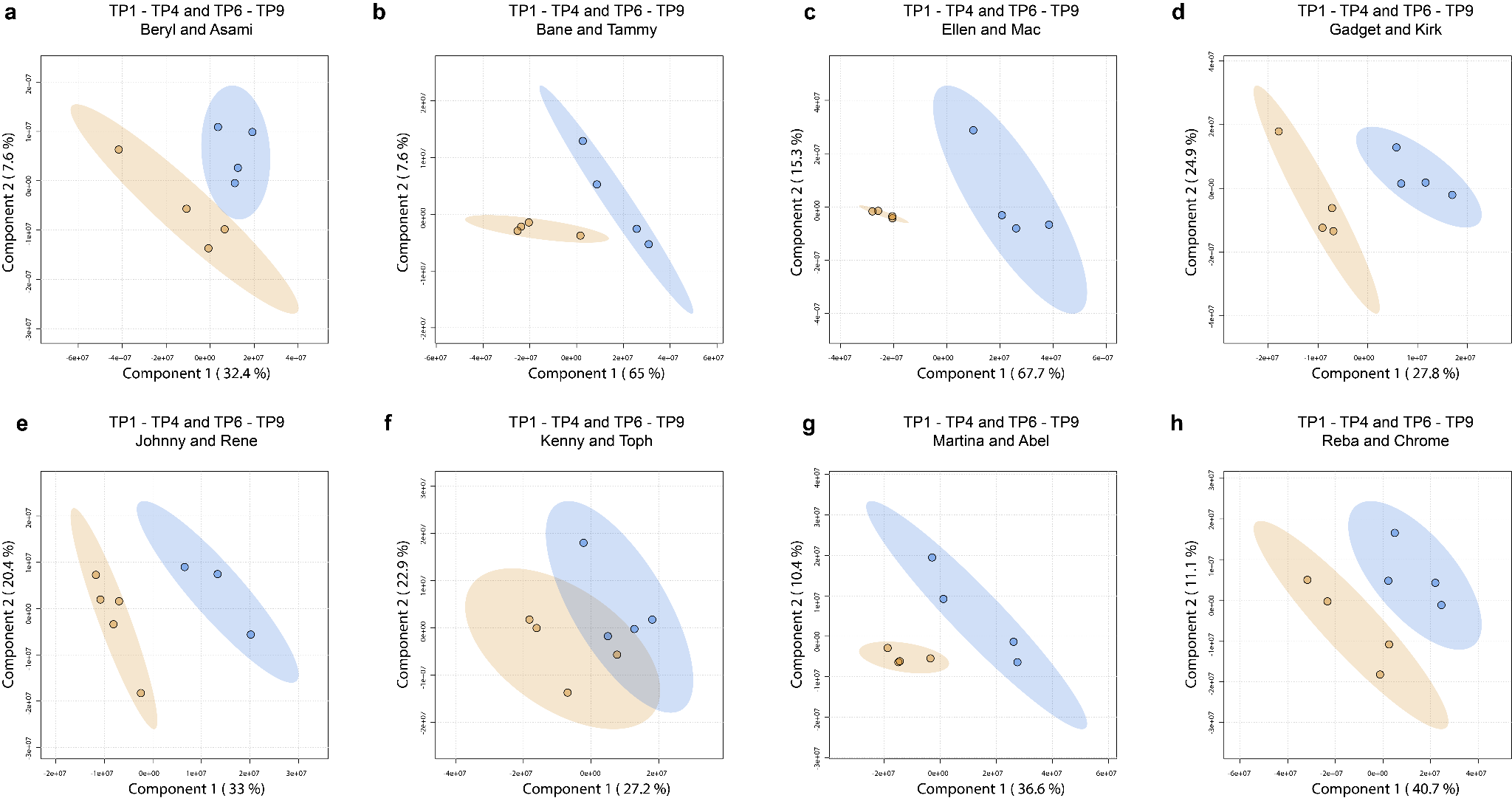


**Supplemental Figure 4.** (**a-h**) Separate PLS-DA analysis of fecal samples by pair of marmosets. Transition time point was excluded for this analysis. Identification of molecules of interest was performed using spectral similarity to reference libraries in the GNPS platform.

**Supplemental Table 1:** This file contains all fecal metabolites for all timepoints. contains 80 (samples) by 3392 (peaks(mz/rt)) data matrix.

**Supplemental Table 2:** This file contains fecal metabolite data filtered to contain only the time points TP1-4 (Pre-diet change group) and TP6-9 (Post-diet change group), corresponding to the months just before and after diet change. This file contains 64 (samples) by 2007 (peaks(mz/rt)) data matrix.
